# Electrochemical carbonyl reduction on single-site M–N–C catalysts

**DOI:** 10.1038/s42004-023-01008-y

**Published:** 2023-09-30

**Authors:** Wen Ju, Alexander Bagger, Nastaran Ranjbar Saharie, Sebastian Möhle, Jingyi Wang, Frederic Jaouen, Jan Rossmeisl, Peter Strasser

**Affiliations:** 1 Chemical Engineering Division, Department of Chemistry, Technical University Berlin, Berlin, Germany; 2 Department of Physics, Technical University of Denmark, Lyngby, Denmark; 3 Institute Charles Gerhardt Montpellier, Univ. Montpellier, CNRS, ENSCM, Montpellier, France; 4 Department of Chemistry, University Copenhagen, Universitetsparken 5, 2100 Copenhagen, Denmark

**Keywords:** Electrocatalysis, Sustainability, Carbon capture and storage, Electrocatalysis, Computational chemistry

## Abstract

Electrochemical conversion of organic compounds holds promise for advancing sustainable synthesis and catalysis. This study explored electrochemical carbonyl hydrogenation on single-site M–N–C (Metal Nitrogen-doped Carbon) catalysts using formaldehyde, acetaldehyde, and acetone as model reactants. We strive to correlate and understand the selectivity dependence on the nature of the metal centers. Density Functional Theory calculations revealed similar binding energetics for carbonyl groups through oxygen-down or carbon-down adsorption due to oxygen and carbon scaling. Fe–N–C exhibited specific oxyphilicity and could selectively reduce aldehydes to hydrocarbons. By contrast, the carbophilic Co–N–C selectively converted acetaldehyde and acetone to ethanol and 2-propanol, respectively. We claim that the oxyphilicity of the active sites and consequent adsorption geometry (oxygen-down vs. carbon-down) are crucial in controlling product selectivity. These findings offer mechanistic insights into electrochemical carbonyl hydrogenation and can guide the development of efficient and sustainable electrocatalytic valorization of biomass-derived compounds.

## Abstract (web summary)

Electrochemical reduction of carbonyl groups is a promising sustainable route for converting biomass-based compounds into value-added products. Here, the authors investigate the electrochemical reduction of the model reactants formaldehyde, acetaldehyde, and acetone on single-site M–N–C catalysts (M = Fe, Co and Ni), gaining mechanistic insight into the role that the nature of the metal center plays on the selectivity of these carbonyl reduction reactions.

## Introduction

Electrochemical catalysis has the potential to revolutionize organic synthesis by creating high-value products using electricity. By utilizing voltage and current, it is possible to bypass the need for high temperature, pressure, and stoichiometric amounts of redox agents, directly altering the functional groups’ structure and oxidation state^1–5^. In recent years, the increasing adoption of renewable energy has made green electricity more accessible and affordable^6^, leading to growing interest in coupling electrolysis technologies with novel synthesis approaches. The combination of electrocatalysis and bio-derived compounds shows great promise for improving both the technological and economic aspects of organic and green chemical synthesis, paving the way for a more sustainable future for green chemistry^7^.

Carbonyl groups received significant attention due to their wide existence in electrochemical biomass and CO_2_ valorization approaches^8–26^. The carbonyl group can be electrochemically oxidized to carboxylates or reduced to oxygenates and alkanes^20,23,27^. Previously, we showed that formaldehyde could be reduced to alcohols on metals^22^. However, these electro-organic reactions are catalyst-dependent and involve complicated proton-coupled and -decoupled reaction steps, making detailed mechanistic understanding at the atomic scale elusive.

**Fig. 1:**
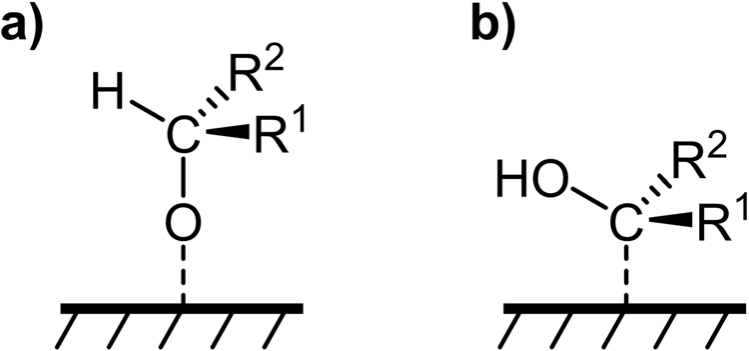
Speculated reaction cascade of carbonyl reduction on the catalyst surface. **a** Adsorption in oxygen-down form and **b** adsorption in carbon-down form. R_1_ and R_2_ represent the alkyl group or H.

Figure 1 depicts generalized steric configurations of how a carbonyl group can be adsorbed on the catalyst surface. At negative potentials, adding a proton-electron pair or hydrogen to the C=O group, the initial reaction intermediate adsorption can occur through oxygen binding (Fig. 1a) or carbon binding (Fig. 1b), each leading to various channels and products. Following Fig. 1a (oxygen-down form), Eley-Rideal hydrogenation on the unbound carbon atom can split the C-O bond, producing alkanes, such as formaldehyde reduction on single-site Fe–N–C candidates^26^. Alternatively, Langmuir-Hinshelwood hydrogenation on the “oxygen-down” intermediate may yield alcohols on metals^22–24^. Additionally, in the case of the initial horizontal two-site adsorption type as proposed in ketone reduction on Pt facets, strong binding energy can dissociate the C=O bond and fully protonate the intermediate to hydrocarbons. However, this adsorption type is not considered on these M–N–C catalysts. In contrast, weak adsorption leads to oxygenate formation^19,20^. These reduction mechanisms contain multiple possibilities and remain unaddressed.

Herein, we investigated the electrochemical reduction of carbonyl groups on single-site M–N–C catalysts (M: Fe, Co, and Ni) using formaldehyde, acetaldehyde, and acetone as model reactants. We strive to establish correlations between the nature of the metal center, adsorption geometry (oxygen-down vs carbon-down), and the products selectivity (hydrocarbons vs oxygenates). As the scaling relations link oxygen-down ($\Delta {E}_{{CH}3O* }=\Delta {E}_{{CH}3{CH}2O* }=\Delta {E}_{{CH}3{CHO}* {CH}3}$) and carbon-down ($\Delta {E}_{* {CH}2{OH}}=\Delta {E}_{{CH}3* {CHOH}}=\Delta {E}_{{CH}3* {COHCH}3}$) adsorption configurations according to our Density Functional Theory calculation, our findings highlight the critical role played by the metal center’s oxyphilicity and the resulting adsorption geometry in controlling product selectivity. Specifically, Fe–N–C exhibited oxyphilic behavior, leading to oxygen-down adsorption for carbonyl groups and enhancing the selectivity for hydrocarbons formation during aldehyde group reduction. Conversely, carbophilic Co–N–C exhibited the ability to adsorb the carbon site of carbonyl groups in acetaldehyde and acetone, yielding ethanol and 2-propanol, respectively.

## Results

### Density functional theory predicted adsorption geometry

In this work, we study the electrocatalytic reduction of carbonyl compounds using a set of single-site M–N–C as model catalysts. The M–N–C catalysts feature isolated single metal atoms with nitrogen coordination, forcing sole atom adsorption (displayed in Fig. 1a, b). We selected formaldehyde, acetaldehyde, and acetone as reactants (C1–C3 carbonyls) for the electrochemical reduction reaction^22^. By varying the metal center of the active M–N_x_ motifs, the binding strength to the reactive intermediates (C vs. O) can be tuned, resulting in distinct reaction paths and corresponding selectivity. Our framework utilizes the adsorption geometry of the carbonyl group (oxygen-down vs. carbon-down) and the binding strength as the selectivity-determining indicator for the production of alcohols or alkanes.

**Fig. 2:**
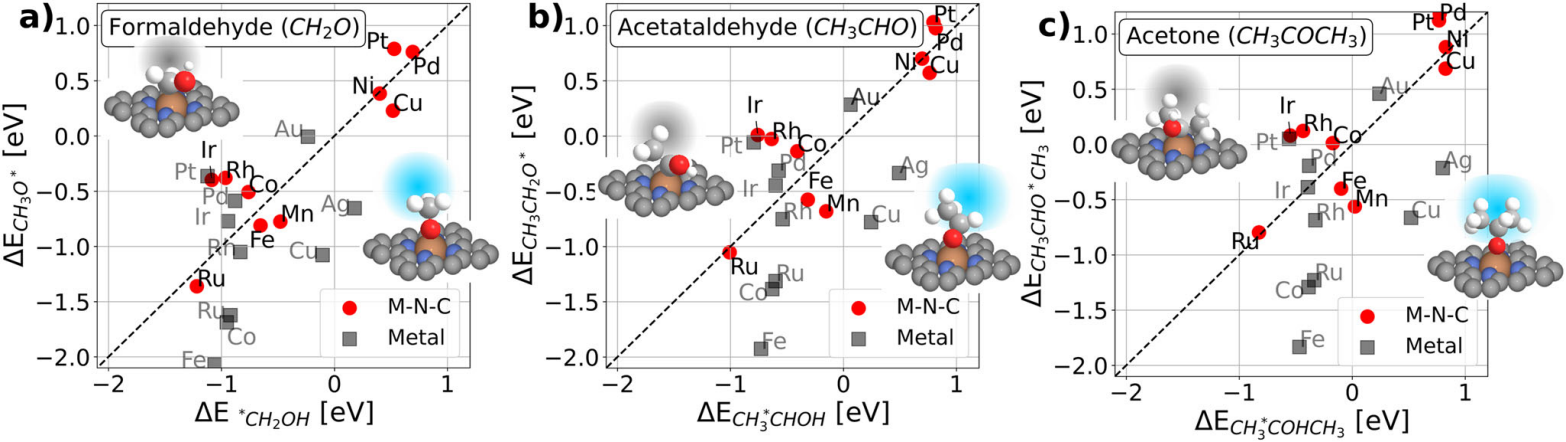
Carbonyl reduction intermediates. **a** Formaldehyde (CH_2_O), **b** acetaldehyde (CH_3_CHO), and **c** acetone (CH_3_COCH_3_) with the oxygen-bound intermediates on the *y*-axis and carbon-bound intermediates on the *x*-axis. While M–N–C’s catalyst (red cycle) falls close around the diagonal, metal catalysts (gray cubic) are more scattered, with some having stronger oxygen binding.

Density Functional Theory was used to determine the binding energies of the initial adsorbed carbonyl intermediates after a proton–electron transfer step, in both carbon-down and oxygen-down forms. Figure 2 illustrates the binding strengths of these two adsorption configurations for various M–N_x_ site’s structure (red) and metal (111) surfaces (black), featuring the carbonyl groups ranging from C1 to C3: formaldehyde (Fig. 2a), acetaldehyde (Fig. 2b), and acetone (Fig. 2c). The diagonal in all panels represents balanced adsorption energy line of the oxygen-down and carbon-down forms. Regions below the diagonal line favor oxygen-down adsorption configuration, while the upper regions prefer carbon-down adsorption (schematic illustrations are detailed in all panels). An important observation is that the binding energy patterns in a, b, and c are similar due to scaling relations between oxygen intermediates: (CH3O*, CH3CH2O*, and CH3CHO*CH3), which all have one oxygen bond to the catalyst surface and between carbon intermediates: (*CH2OH, CH3*CHOH and CH3*COHCH3), which all have one carbon bond to the surface. The simplistic picture is general, with one oxygen versus one carbon bond as represented for OH* vs. *CH3 shown in Fig. S1. The origin of the fundamental scaling relation between the oxygen bonds and carbon bonds can be shown for both M–N–C catalyst and for metal catalysts as depicted in Fig. S2.

Focusing on Fig. 2, we notice the theoretical perspective that most metallic catalysts exhibit a preference for binding with oxygen-down over carbon-down (Fe, Co, Ru, Cu, and Ag), except for precious metals like Pt, Au, Rh, and Pd, which are close to the diagonal. In contrast, most M–N–C candidates exhibit similar binding strengths for both adsorptions, positioned close to the diagonal line. Our benchmark study of M–N–C s showed that Co–N–C is located in the carbon-down region, while Fe–N–C is slightly in the oxygen-down region for all projected carbonyl compounds, e.g., the difference in formaldehyde carbon binding is 0.16 eV (Co–N–C: −0.76 eV, Fe–N–C: −0.6 eV) and oxygen binding is 0.3 eV (Co–N–C: −0.51 eV, Fe–N–C: −0.81 eV) for Co–N–C and Fe–N–C. This difference could potentially provide a different reaction path on our two selected catalysts. In line with previous work on oxygen reduction reaction, Fe–N–C allows the four-electron reduction of oxygen to water, whereas the weaker oxyphilic Co–N–C site is more selective toward the two-electron reduction toward H_2_O_2_^28^. This suggests Fe–N–C’s capability to bind oxygen (O*) at the active site, whereas this is partly limited to Co–N–C. On Ni–N–C, both carbon-down and oxygen-down exhibit similar but weak binding energies for intermediate adsorption.

### Electrochemical reduction of carbonyl groups on M–N–C catalysts

We conducted electrochemical reduction experiments on Fe–N–C, Co–N–C, and Ni–N–C catalysts derived from ZIF-8. Those candidates have been shown to possess single metal atoms as active sites, and their exposed surface area and metal concentration remain in a similar range (Supplementary Information Table S1, Fig. S3 and Note)^29^. A reference candidate without our studied metal centers in this work is named N–C. All reactants selected in this set study are CH_2_O, CH_3_CHO, and CH_3_COCH_3_, with an initial concentration of 5 mM.

We first analyzed the behavior of formaldehyde reduction by performing linear sweep amperometry (LSV) from −0.2 to −0.8 V_RHE_ before the bulk electrolysis and measuring the geometric current density as a function of scanning potential (Fig. S4). The LSV plots of N–C, Fe–N–C, and Ni–N–C catalysts showed only minor differences with the presence of CH_2_O. Interestingly, the Co-based catalyst seems poisoned by CH_2_O (Fig. S4a).

Later, we held the cathode potential stationary for 75 min for product analysis (details are given in “Method” section and Supplementary Information). The hydrogen evolution reaction was the prominent process, leaving only <10% faradaic efficiency for CH_2_O conversion. Moreover, within 75 min of electrolysis, other liquid products, such as CH_3_OH, were not detectable. This latter observation is different from CH_2_O reduction on metal catalysts^22^.

**Fig. 3:**
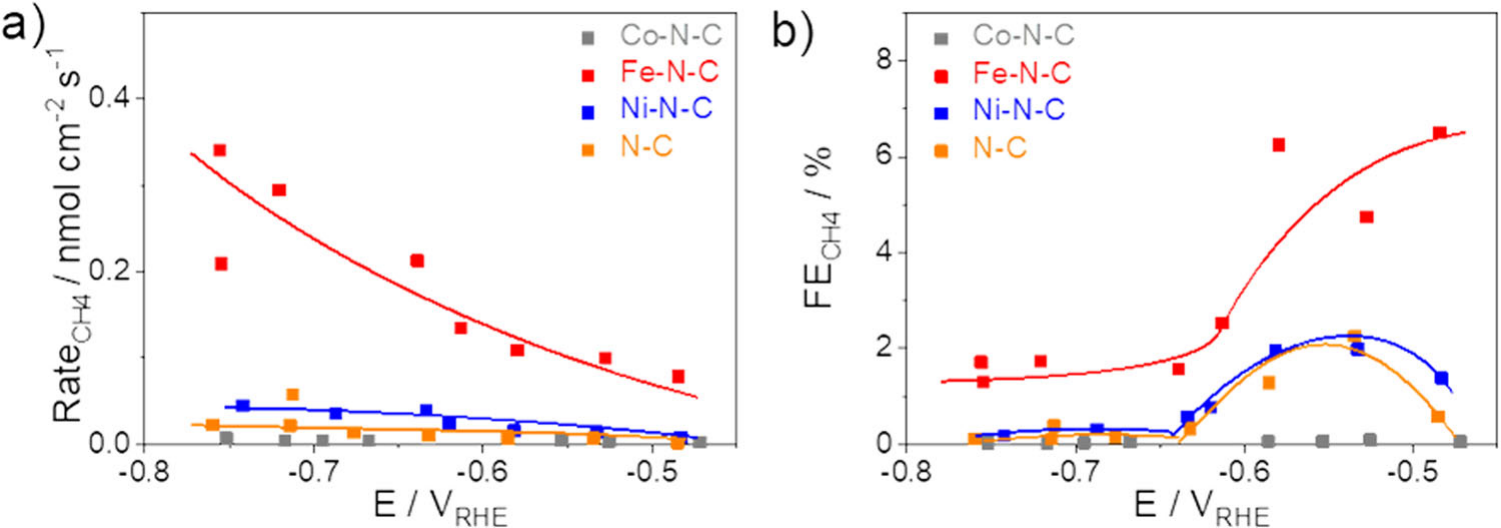
Reactivity of electrochemical CH_2_O reduction. **a** CH_4_ production rate and **b** CH_4_ faradaic efficiency as a function of the applied iR-free electrode potential on N–C (orange), Fe–N–C (red), Co–N–C (gray), and Ni–N–C (blue) catalysts. Data are averages over 75 min electrolysis obtained at 15 min, 45 min, and 75 min of each electrolysis. Line to guide the eye. Conditions: 0.05 M K_3_PO_4_ + 0.05 M H_3_PO_4_ neutral solution. Catalyst loading: 0.75 mg cm^−2^ on glassy carbon. The polarization curve of CH_2_O reduction is shown in Fig. S4 and pH dependence of CH_2_O reduction is presented in Fig. S5.

Figure 3a, b shows the reaction rate and faradaic efficiency of CH_4_ formation, highlighting the role of the nature of the metal centers for the reactivity of CH_2_O reduction. Fe–N–C yielded CH_4_ with a maximum efficiency of 6% at −0.5 V_RHE_. The reaction rate increased with more cathodic potentials while the faradaic efficiency decreased. On the contrary, Co–N–C was entirely inactive for CH_2_O to CH_4_ conversion. The LSV profile evidenced that CH_2_O poisoned Co–Nx sites and thus deactivated the catalyst (Fig. S4). The reference N–C catalyst showed a very low, yet, in a certain potential range, finite CH_4_ formation. We hypothesize that this minor CH_4_ reactivity can be attributed to the presence of distinct nitrogen species or residual Zn metal atoms.

**Fig. 4:**
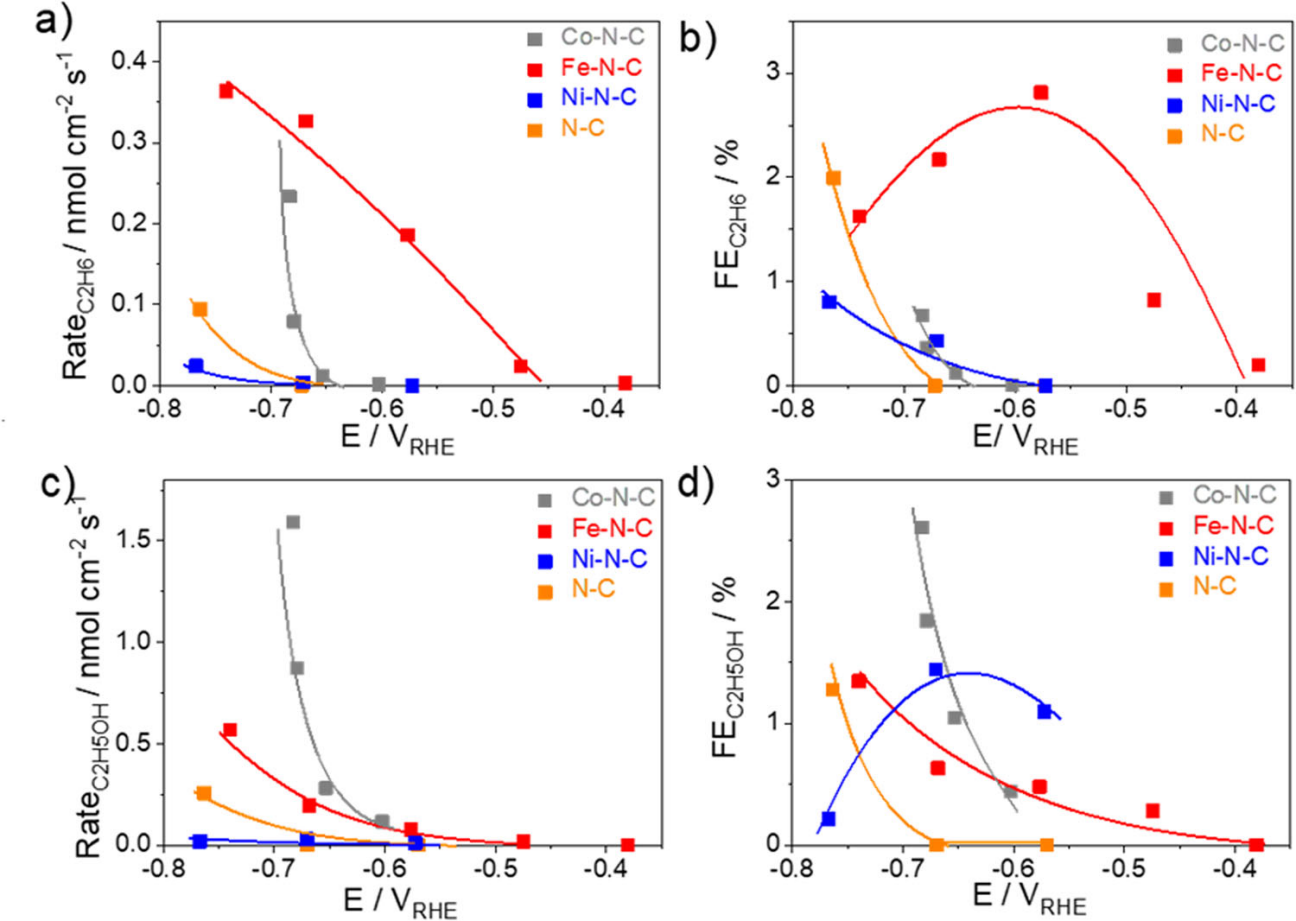
Reactivity of electrochemical CH_3_CHO reduction. **a** C_2_H_6_ production rate, **b** C_2_H_6_ faradaic efficiency, **c** C_2_H_5_OH production rate, and **d** C_2_H_5_OH faradaic efficiency as a function of the applied iR-free electrode potential on N–C (orange), Fe–N–C (red), Co–N–C (gray), and Ni–N–C (blue) catalysts. Data are averages over 75 min electrolysis obtained at 15 min, 45 min, and 75 min of each electrolysis. Line to guide the eye. Conditions: 0.05 M K_3_PO_4_ + 0.05 M H_3_PO_4_ neutral solution. Catalyst loading: 0.75 mg cm^−2^ on glassy carbon. The polarization curve of CH_3_CHO reduction is shown in Fig. S6 and pH dependence study is presented in Fig. S7.

We then investigated the reduction of CH_3_CHO on these single-site catalysts and found that C_2_H_6_ and C_2_H_5_OH achieve roughly 3% faradaic efficiency (Fig. 4), whereas the rest of the current majorly contribute to hydrogen evolution. The Ni–N–C catalyst was inactive, yielding only a low production rate (<0.1 nmol cm^−2^ s^−1^) at ~−0.8 V_RHE_. In addition, the Fe–N–C catalyst exhibited a higher selectivity for C_2_H_6_ formation, with the onset at −0.5 V_RHE_ and a peak faradaic efficiency of 3% at −0.6 V_RHE_. This aligns with the observed preference for oxygen-down binding and protonation on the unabsorbed site, as seen in the CH_2_O reduction to CH_4_. On the other hand, the Co–N–C catalyst produces C_2_H_5_OH with a reaction rate of 1.5 nmol cm^−2^ s^−1^ and a faradaic efficiency of 3% at −0.7 V_RHE_. Notably, this differs from experimental observation for CH_2_O reduction in that only hydrocarbons are observed on Fe–N–C and no product from Co–N–C, which could be plausibly attributed to the Keto-Enol tautomerism of acetaldehyde molecule.

**Fig. 5:**
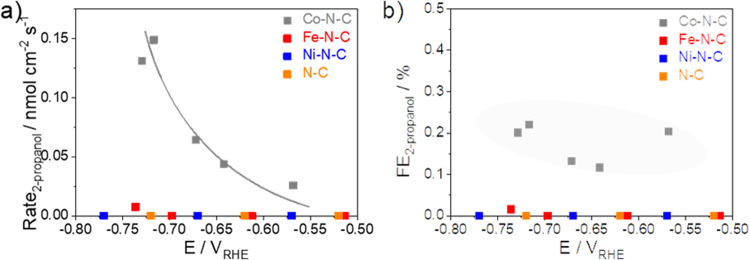
Reactivity of electrochemical CH_3_COCH_3_ reduction. **a** 2-propanol production rate and **b** 2-propanol faradaic efficiency as a function of the applied iR-free electrode potential on N–C (orange), Fe–N–C (red), Co–N–C (gray), and Ni–N–C (blue) catalysts. Data are averages over 75 min electrolysis obtained at 15 min, 45 min, and 75 min of each electrolysis. Line to guide the eye. Conditions: 0.05 M K_3_PO_4_ + 0.05 M H_3_PO_4_ neutral solution. Catalyst loading: 0.75 mg cm^−2^ on glassy carbon. The polarization curve of CH_3_COCH_3_ reduction is shown in Fig. S8.

In our final investigation, we focused on the electrochemical reduction of CH_3_COCH_3_ on M–N–C candidates (Fig. 5). Only the Co–N–C catalyst showed a measurable electrochemical reduction of CH_3_COCH_3_ to 2-propanol. The catalytic conversion started at around −0.5 V_RHE_ and increased with more negative potentials, reaching a maximum production rate of 1.5 nmol cm^−2^ s^−1^. However, the faradaic efficiency for this reaction remained low at around 0.2% in our studied potential range.

## Discussion

In summary, our study employed a combination of theoretical and experimental approaches to screening electrochemical carbonyl reduction on single-site M–N–C catalysts, highlighting the crucial role of the adsorption geometry of the carbonyl group in determining product selectivity. Unlike the metal surfaces (oxygen-down adsorption selectively leads to oxygenates formation, summarized in Table 1), the oxyphilic Fe-functionalized active site preferentially binds to the oxygen site of aldehyde groups (Fig. 1a, oxygen-down adsorption) and exhibits significant faradaic efficiencies towards CH_4_ and C_2_H_6_ (8% and 3%, respectively). Notably, during the acetaldehyde reduction, C_2_H_5_OH arises as a by-product at more negative potentials, plausibly through a specific reaction mechanism after the initial Keto-Enol tautomerism. For acetone, the steric structure and I-effect from the two methyl groups pose a hindrance to the overall reaction reactivity. As a result, through predicted oxygen-down adsorption, the Fe–N–C catalyst yields neither hydrocarbons nor oxygenates during the ketone group reduction. On the contrary, the carbophilic (less oxyphilic) Co–N–C, according to our prediction, prefers carbon binding, selectively converting acetaldehyde and acetone to oxygenates (or being poisoned by formaldehyde). This finding demonstrates a ketone-to-oxygenate path through the carbon-down adsorption geometry. Overall, our insights into carbon-based molecule conversions on single-site catalysts could contribute to a better fundamental understanding of electrochemical CO_2_ and biomass valorization reactions and could pave the way for potential large-scale electrochemical carbonyl hydrogenation processes.

**Table 1:** Summary of product selectivity of electrochemical carbonyl reaction on our studied single site M–N–C catalysts in combination with DFT predicted initial adsorption geometry.

Catalyst		Reactant	Predicted adsorption geometry^a^	Hydrocarbons	Oxygenates
Metal	Cu	CH_2_O^22^_(1 mM)_	O-down	No	Yes
	Ag		O-down	No	Yes
	Au		C/O-down	Minor	Minor
	Cu	CH_2_O^33^_(50 mM)_	O-down	Minor	Yes
	Cu	CH_3_CHO^23,24^	O-down	No	Yes
	Pt(553)	CH_3_COCH_3_^20^	C-down	No	Yes
	Pt(510)		C-down	Yes	No
Solid-state M–N–C catalyst (This work)	Fe–N–C^b^	CH_2_O	O-down	Yes	No
	Co–N–C		C-down	No (Poison)	No (Poison)
	Ni–N–C		C/O-down	No	No
	Fe–N–C	CH_3_CHO	O-down	Yes	Minor
	Co–N–C		C-down	Minor	Yes
	Ni–N–C		C/O-down	No	No
	Fe–N–C	CH_3_COCH_3_	O-down	No	No
	Co–N–C		C-down	No	Minor
	Ni–N–C		C/O-down	No	No

^a^Adsorption geometry follows the theoretical prediction in this work.

^b^Experimental phenomena are identical to our previous study on similar Fe–N–C catalyst^26^.

## Methods

The catalyst synthesis is identical to our previous work^29^ and the protocol is detailed in the Supplementary Information. For catalyst ink, 15 mg catalyst powder was first mixed with 50 μL Nafion solution (5 wt% solved in ethanol solution, SigmaAldrich), 150 μL isopropanol, and 800 μL DI-water, and later sonicated using SoniferHorn for 15 min. 50 μL catalyst ink was later drop casted on 1 cm^2^ glassy carbon plate and dried to our working electrode, for a catalyst loading of 0.75 mg cm^−2^.

All electrochemical assessments were conducted in a custom-made two-compartment and three-electrode H-cell, using EC-Lab SP-200 Potentiostat. The cathode chamber and anode chamber were separated using a Nafion 117 membrane. Pt mesh was deployed as the counter anode. A leak-free Ag/AgCl was used as the reference, located in the cathode chamber, close to our working electrode.

5 mM carbonyl compounds, namely, the CH_2_O, CH_3_CHO, and CH_3_COCH_3_, were added in N_2_-purged (30 sccm) KH_2_PO_4_/K_2_HPO_4_ (0.1 M anion concentration, Sigma-Aldrich) neutral buffer electrolyte as reactants. For measurements, the impedance between reference electrode and working electrode was measured with Potential Electrochemical Impedance Spectroscopy (PEIS) at −1.0 V vs Reference. The measurements were controlled using constant potential with 50% automatic IR correction, whereas the rest were manually corrected. Gas products were detected by an online Gas Chromatograph and the residual electrolyte were analyzed using HPLC and liquid Gas Chromatograph for liquid products. Quantification details are given in the Supplementary Information and Supplementary Eqs. (1)–(4).

For Density Functional Density calculation, the M–N–C model was created in ASE^30^ by a 3 × 5 unit graphene cell with a functionalized M-N_4_ site by removing carbon atoms. The outmost carbon atoms were fixed in position and periodic boundaries were applied. Further, the metal (111) model was built by a standard 3 × 3 × 4 slab including a vacuum region and the two lower layers fixed. The electronic calculations were carried out with the GPAW software^31^ with the projector augmented wave method, spin polarization and the revised Perdew–Burke–Ernzerhof functional^32^. We applied a 0.18 grid spacing together with a (2 × 2 × 1) k-point sampling for M-N-Cs and (3 × 3 × 1) k-point sampling for the metals and all the structures were relaxed to a force below 0.1 eV/Å.

### Supplementary information

Supplementary Information

Description of Additional Supplementary Files

Supplementary Data 1

## Data Availability

All experimental electrochemical data are given in Supplementary Data 1 and other data can be obtained from the authors upon a reasonable request.
