# Supplementary material for: Electrochemical carbonyl reduction on single-site M–N–C catalysts
Source: Commun Chem. 2023 Sep 30;6:212. doi: 10.1038/s42004-023-01008-y (PMC10542751; doi:10.1038/s42004-023-01008-y)
Supplement: Supplementary file 1 — Supplementary Information [file 42004_2023_1008_MOESM1_ESM.pdf]

Supporting online information

Electrochemical carbonyl reduction on single-site M-N-C catalysts

Wen Ju,[1+] Alexander Bagger,[2+] Nastaran Ranjbar Saharie,[3] Sebastian Möhle,[1] Jingyi Wang,[1] Frederic Jaouen,[3] Jan Rossmeisl,[4] and Peter Strasser[1]*

1. Chemical Engineering Division, Department of Chemistry, Technical University Berlin, Berlin, Germany
2. Department of Physics, Technical University of Denmark, Lyngby, Denmark
3. Institute Charles Gerhardt Montpellier, Univ. Montpellier, CNRS, ENSCM, Montpellier, France
4. Department of Chemistry, University Copenhagen, Universitetsparken 5, 2100 Copenhagen

Wen Ju and Alexander Bagger contributed equally.

Corresponding author:

Peter Strasser pstrasser@tu-berlin.de

Contents

Electrochemical carbonyl reduction on single-site MNC catalysts 1

Supplementary Methods 3

Catalysts Synthesis 3

Products Analysis 3

Supporting DFT Data 5

Part of ex-situ Physicochemical Characterizations 6

Supporting Electrochemical Data 8

Linear Sweep Amperometry of CH_2O reduction 8

pH study of CH_2O reduction 9

Linear Sweep Amperometry of CH_3CHO reduction 10

pH study of CH_3CHO reduction 11

Linear Sweep Amperometry of CH_3COCH_3 reduction 12

Supplementary References: 13

Supplementary Methods

Catalysts Synthesis

The single-site M-N-C catalysts deployed in this work were taken from our previous work.[1] All candidates were prepared via dry ball-milling of the Zn(II) zeolitic imidazolate framework (Basolite Z1200 from BASF, labeled ZIF-8), M^{2+} acetate (M = Fe, Co, and Ni), and 1,10-phenanthroline. The catalyst precursors from the milling were pyrolyzed at 1050 °C in Ar for 1 h. The obtained catalysts were labeled M-N-C (Co, Fe, Ni). A control sample was also synthesized according to this process, except that no separate metal acetate was added. Considering the Zn evaporation at over 1000 °C, we label this N-C.

Products Analysis

Gas samples were analyzed with an Online Gas Chromatograph (Shimadzu GC 2014) equipped with a thermal conductivity detector (TCD) and a flame ionization detector (FID). Argon (Air liquid 5.0) was employed as carrier gas. The gaseous compounds H_2, N_2, O_2, CH_4 and CO were separated in a HayeSep column (HayeSep Q + HayeSep R). Gas samples were tested at 15 min, 45 min and 75 min of each electrolysis, and the data are averages of these three-time points. Reaction rate and faradaic efficiency of gas products are calculated using Supplementary Equation 1 and 2.

Liquid product formate (not detectable in this framework) was analyzed by high performance liquid chromatograph (Agilent 1200 series) equipped with an organic acid resin from Ziemer chromatography ® column, a reflection index detector (RID). The alcohol products were analyzed by liquid-injection Gas Chromatograph (Shimadzu 2010 plus) equipped with an OptimaWax column. The liquid sample was first evaporated at 180 ºC, carried by Helium flow, and detected with FID. 2 mL residual electrolyte after electrolysis were collected as a liquid sample.

For electrochemical reduction of CH_2O, formaldehyde solution (37 wt%, Sigma-Aldrich, stabilized with 10% methanol) was added into the phosphate electrolyte. In the presence of CH_3OH, we detected the methanol concentration before and after the reaction to quantify the concentration change (ΔC, shown below in equation S3) according to the reaction. (Note: We did not observe obvious methanol and formaldehyde evaporation in terms of 75 min electrolysis with 20 ccm N_2 bubbling during the reaction at open circle potential.) Acetaldehyde and acetone used in this work has a purity of > 99.5% (Sigma-Aldrich). Reaction rate and faradaic efficiency of gas products are calculated using Supplementary Equation 3 and 4.

Supplementary Equation S1. Production Rate of Gas Products

$$Rate(g) = \frac{\dot{V} * C}{A * V_M}$$

Rate(g): Generation rate of the gas product / mol s^{-1} cm^{-2}

$\dot{V}$: CO_2 gas flow rate / L s^{-1}

C: Concentration of the product detected by GC / Vol%

A: Geometric area of the electrode / cm^2

V_M: Molar volume of ideal gas at ambient condition / 22.4 L mol^{-1}

Supplementary Equation S2. Faradaic Efficiency of Gas Products

$$FE(g) = \frac{Rate(g) * z * F}{j_{total}} * 100\%$$

FE(g): Faradaic Efficiency of the product / %

z: Charge transfer of each product

F: Faradaic Constant / 96500 C mol^{-1}

j_{total}: Total current density during CO_2 bulk electrolysis / A cm^{-2}

Supplementary Equation S3. Reaction Rate of Liquid Products

$$Rate(l) = \frac{V * \Delta C}{t * A}$$

Rate(l): Generation rate of the liquid product / mol s^{-1} cm^{-2}

V: Volume of the electrolyte / L

ΔC: Accumulated concentration of the product detected by HPLC or liquid GC / mol L^{-1}

T: reaction time / s

Supplementary Equation S4. Faradaic Efficiency of Liquid Products

$$FE(l) = \frac{V * \Delta C * z * F}{Q} * 100\%$$

FE(l): Faradaic Efficiency of the liquid product / %

Q: Total charge transfer during the electrolysis / C

Supporting DFT Data

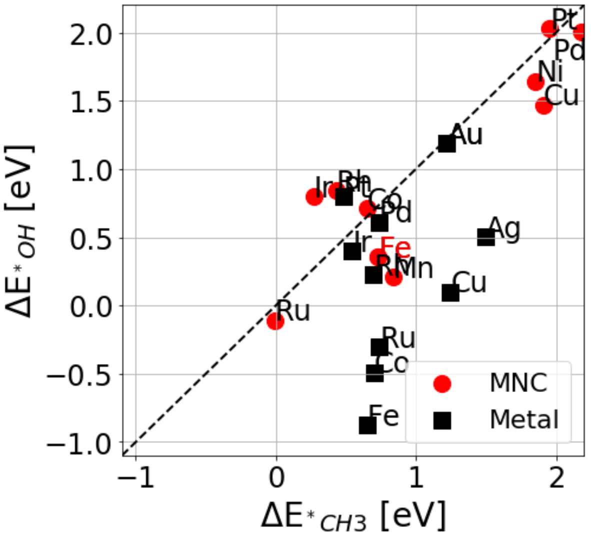

Figure S 1 Reference system: Single oxygen bond (*OH) versus single carbon bond (*CH3) shows an exactly similar image as Figure 2 in main due to the scaling relations.

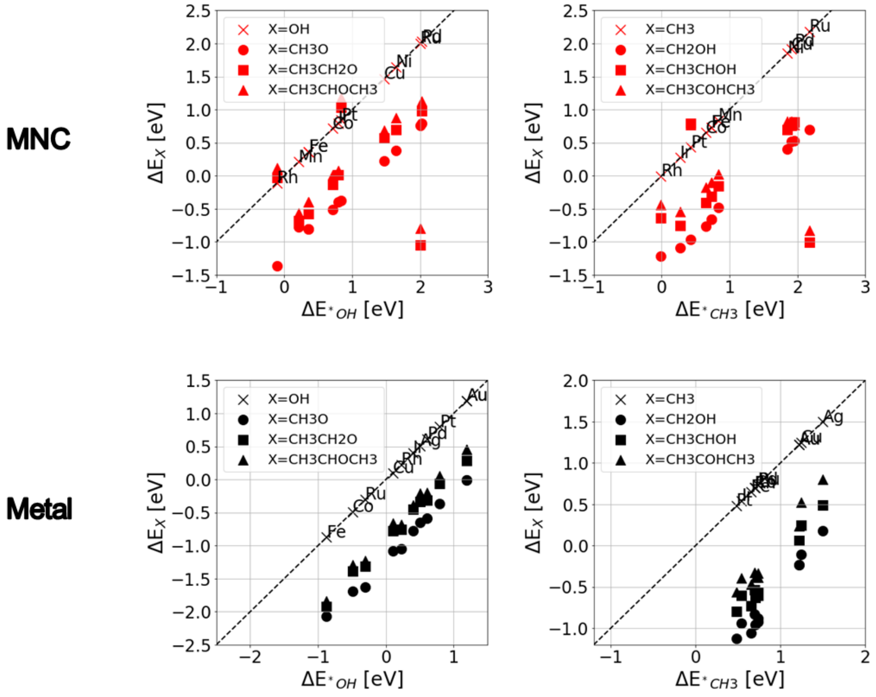

Figure S 2 Depict the scaling relations for M-N-C and Metal catalysts between oxygen intermediates left and carbon intermediates right. On the y-axis the compound X energy is plotted versus either the *OH or *CH3 binding energy. All data fall on a line, showing that the fundamental scaling relations for the intermediates in this study.

Part of ex-situ Physicochemical Characterizations

Table S1. Physical and chemical Characterizations: a) surface element composition analyzed using X-ray photoelectron spectra, b) BET: N_2 ad/desorption-based surface area. Data are adopted from our previous work upon identical catalyst materials.[1]

	**a. Quantitative XPS analysis ($\%_{at.}$)**					**b. BET (m^2 g^{-1})**
	C	N	O	Zn	M	
(Zn)N-C	91.0	4.5	4.4	0.53	--	233
Fe-N-C	89.1	6.6	4.1	0.11	0.18	333
Co-N-C	91.8	4.3	3.6	0.38	0.24	283
Ni-N-C	90.5	4.1	5.3	0.32	0.22	223

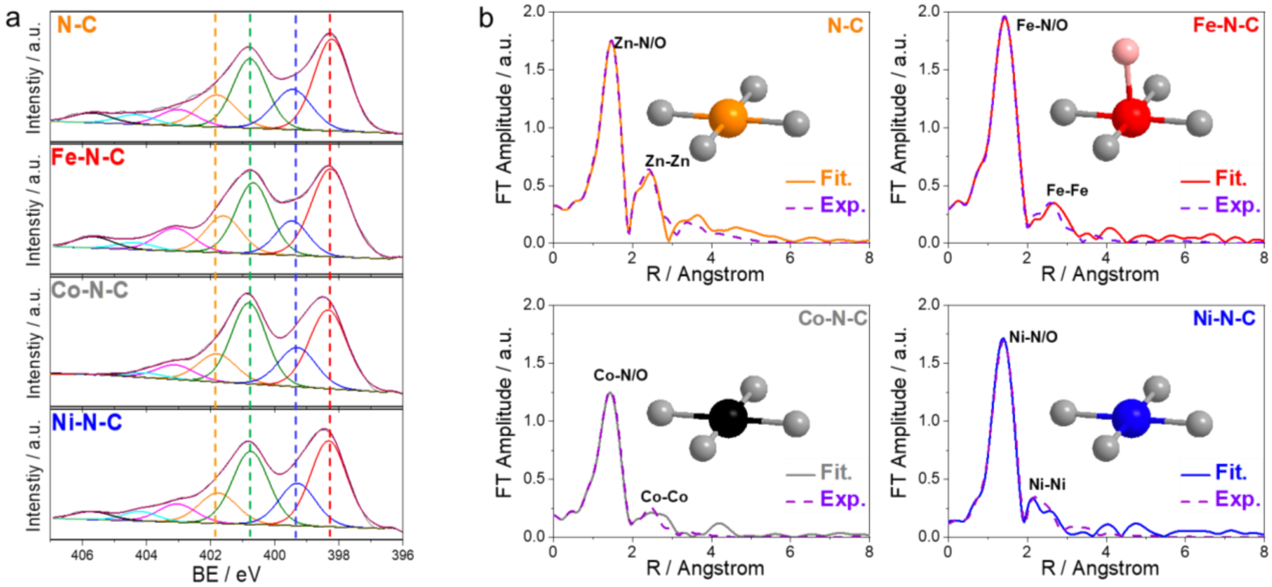

Figure S 3 a) X-ray photoelectron spetra profile of N1s and b) extended X-ray absorption fine structure profiles of our studied catalysts. The catalysts synthesis and regarding ex-situ X-ray based analysis are identical with ref[1].

Note: the N1s profiles of the our studied catalysts are shown in Figure S1a. As fitted, the N group (red) covering the BE (398.1 ± 0.2 eV) to BE (398.7 ± 0.2eV) cover two sp2-hybridized nitrogens bound to carbons, such as imine, pyridinic, or triazinic type N. The group with BE (399.6 ± 0.2 eV, blue) indicates sp2 hybridized N in Metal-N coordination (Ni-N), OC-NH-C partial double bonds, or multiple graphitic N motifs

in a single aromatic ring. Group (401 ± 0.2 eV, green) and V (402.8 ± 0.2 eV, orange) cover the in-plane N-H, graphitic N(-H), and out-of-plane N-H, such as protonated-pyridinic, pyrrolic, graphitic, and quaternary N. Species in groups with BE (> 404 eV) should be assigned to oxide N moieties.[2-5]

The metal K-edge EXAFS spectra were then fitted with the model structures provided in Figure S2 b. For Co-N-C and Ni-N-C, in-plane 4 nitrogen coordinated metal center could be fitted, while the spectra of Fe-N-C was fitted assuming the presence of four in-plane nitrogen atoms with one or two oxygen atoms as axial ligands, in line with the high oxophilicity of Fe.[1]

Supporting Electrochemical Data

Linear Sweep Amperometry of CH_2O reduction

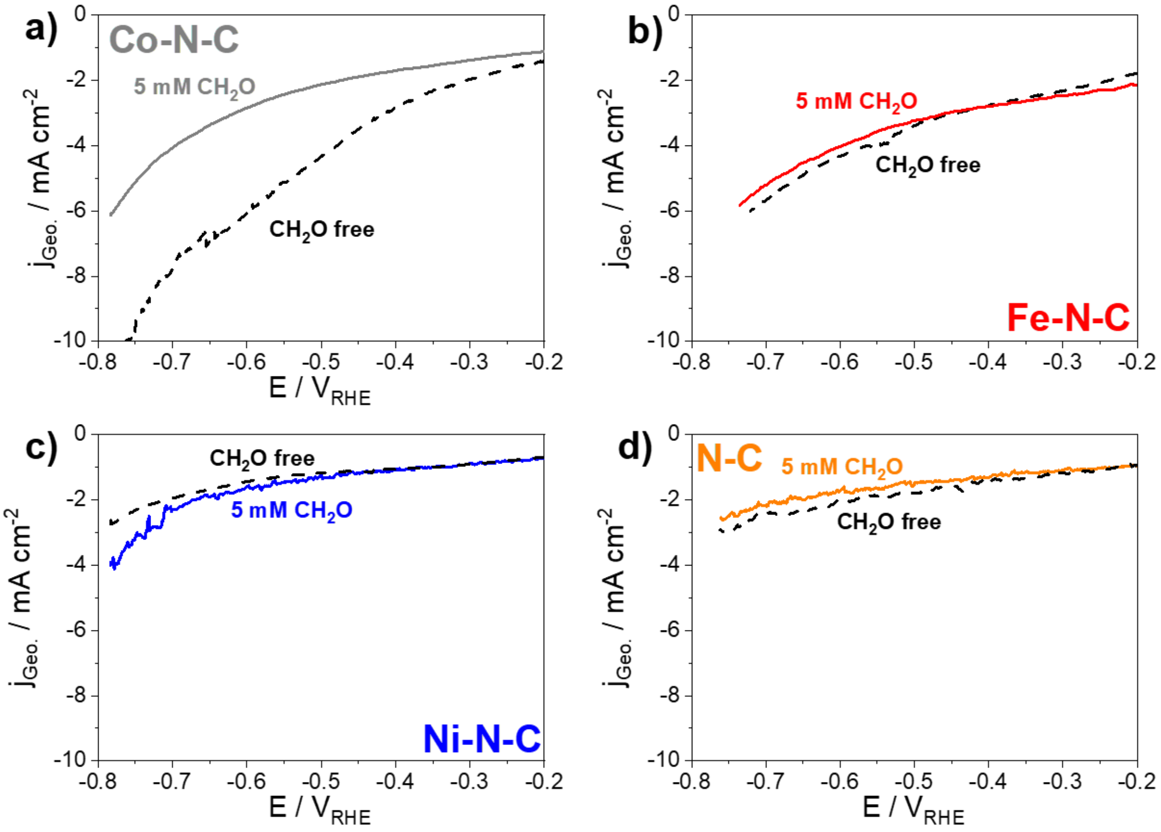

Figure S 4 Polarization curves of CH_2O reduction during cathodic scanning on a) Co-N-C, b) Fe-N-C, c) Ni-N-C, and d) N-C catalysts in neutral potassium phosphate electrolyte (0.05M K_3PO_4 + 0.05M H_3PO_4, pH = 6.9). Solid curves and dash curves display the current densities with and without the presence of CH_2O reactant (concentration: 5 mM), as a function of iR-corrected electrode potentials. Scan rate: -5 mV s^{-1}. Catalyst loading: 0.75 mg cm^{-2} on glassy carbon.

pH study of CH_2O reduction

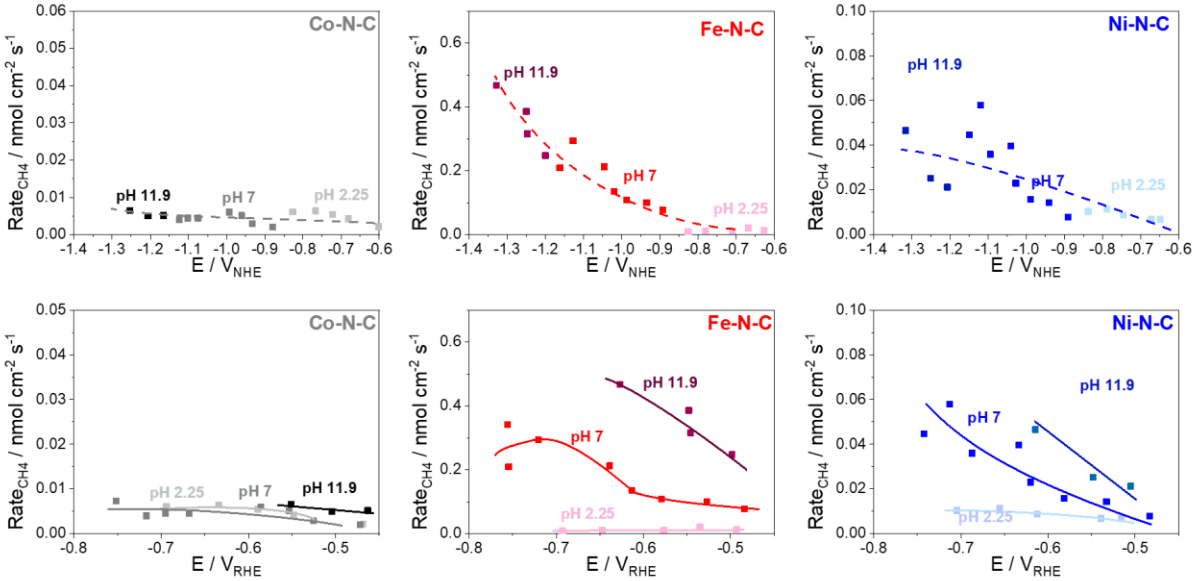

Figure S 5 Electrochemical CH_2O reduction on Co-N-C, Fe-N-C, and Ni-N-C catalysts in different pH conditions (0.05M K_3PO_4 + 0.05M K_2HPO_4, pH = 11.9; 0.05M K_3PO_4 + 0.05M H_3PO_4, pH = 6.9; 0.05M K_2HPO_4 + 0.05M H_3PO_4, pH = 2.25) with initial CH_2O concentration 5 mM, in NHE (upper) and RHE (bottom) scales. Catalyst loading: 0.75 mg cm^{-2} on glassy carbon.

Linear Sweep Amperometry of CH_3CHO reduction

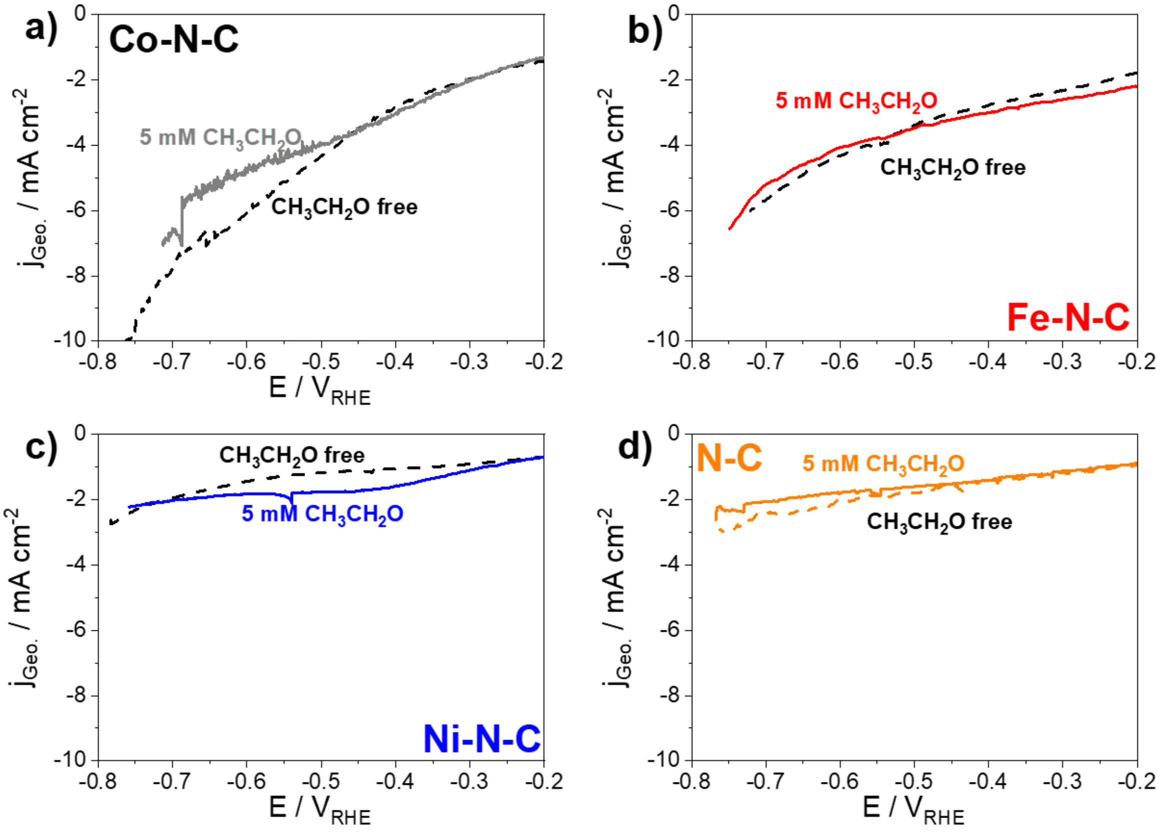

Figure S 6 Polarization curves of CH_3CHO reduction during cathodic scanning on a) Co-N-C, b) Fe-N-C, c) Ni-N-C, and d) N-C catalysts in neutral potassium phosphate electrolyte (0.05M K_3PO_4 + 0.05M H_3PO_4, pH = 6.9). Solid curves and dash curves display the current densities with and without the presence of CH_3CHO reactant (concentration: 5 mM), as a function of iR-corrected electrode potentials. Scan rate: -5 mV s^{-1}. Catalyst loading: 0.75 mg cm^{-2} on glassy carbon.

pH study of CH_3CHO reduction

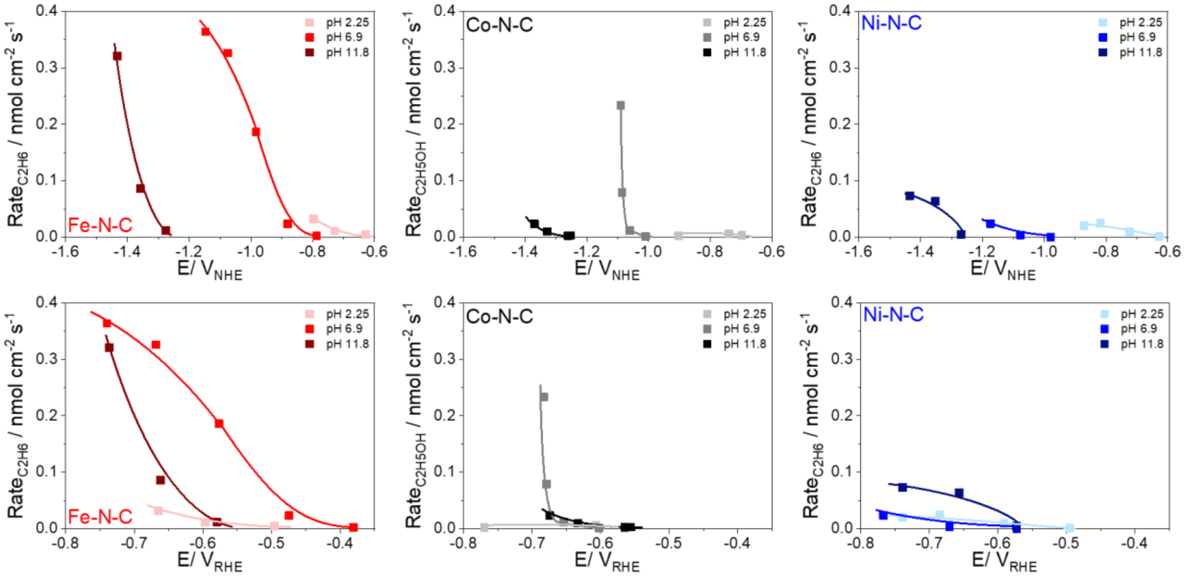

Figure S 7 Electrochemical CH_3CHO reduction on Co-N-C, Fe-N-C, and Ni-N-C catalysts in different pH conditions (0.05M K_3PO_4 + 0.05M K_2HPO_4, pH = 11.9; 0.05M K_3PO_4 + 0.05M H_3PO_4, pH = 6.9; 0.05M K_2HPO_4 + 0.05M H_3PO_4, pH = 2.25) with initial CH_2O concentration 5 mM, in NHE (upper) and RHE (bottom) scales. Catalyst loading: 0.75 mg cm^{-2} on glassy carbon.

Linear Sweep Amperometry of CH_3COCH_3 reduction

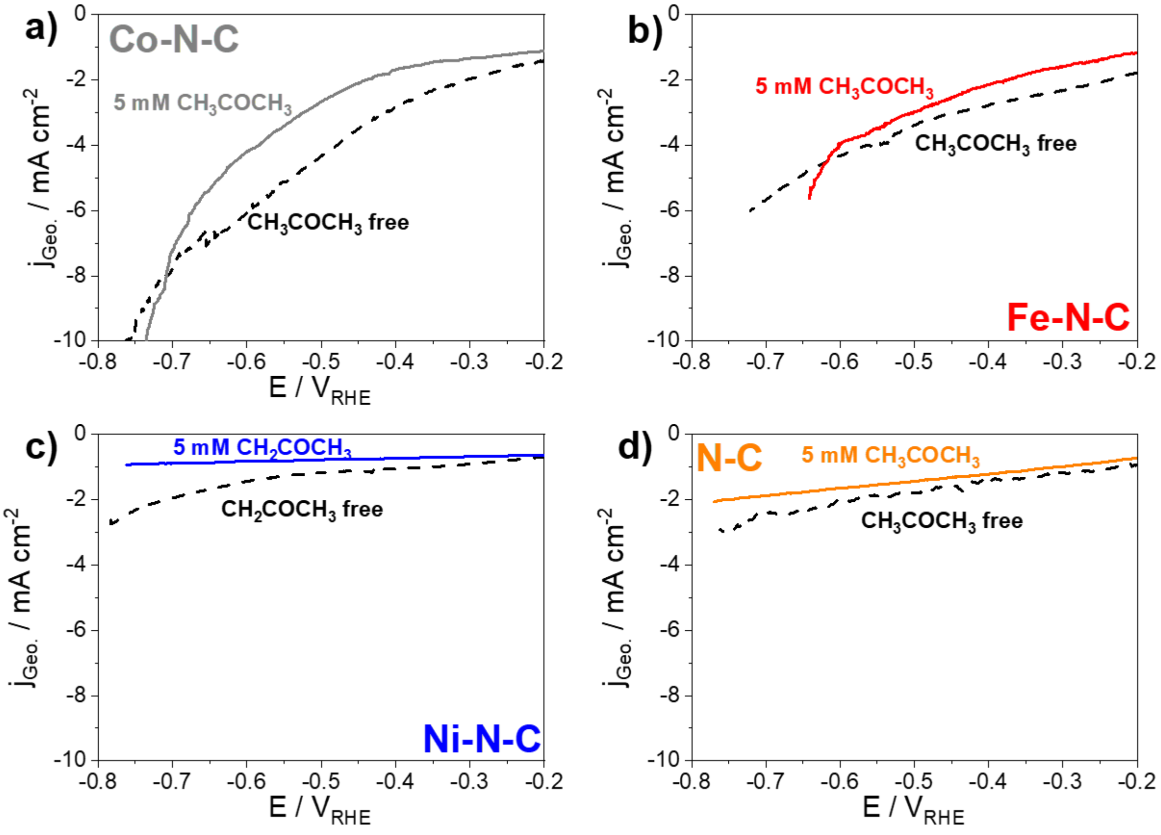

Figure S 8 Polarization curves of CH_3COCH_3 reduction during cathodic scanning on a) Co-N-C, b) Fe-N-C, c) Ni-N-C, and d) N-C catalysts in neutral potassium phosphate electrolyte (0.05M K_3PO_4 + 0.05M H_3PO_4, pH = 6.9). Solid curves and dash curves display the current densities with and without the presence of CH_3COCH_3 reactant (concentration: 5 mM), as a function of iR-corrected electrode potentials. Scan rate: -5 mV s^{-1}. Catalyst loading: 0.75 mg cm^{-2} on glassy carbon.

Supplementary References:

1 Li, J. *et al.* Volcano Trend in Electrocatalytic CO2 Reduction Activity over Atomically Dispersed Metal Sites on Nitrogen-Doped Carbon. *ACS Catalysis* **9**, 10426-10439, doi:10.1021/acscatal.9b02594 (2019).

2 Artyushkova, K. *et al.* Density functional theory calculations of XPS binding energy shift for nitrogen-containing graphene-like structures. *Chem Commun* **49**, 2539-2541, doi:10.1039/c3cc40324f (2013).

3 S. Kabir, K. A., B. Kiefer, P. Atanassov. Computational and experimental evidence for a new TM–N 3/C moiety family in non-PGM electrocatalysts. *Phys. Chem. Chem. Phys.* **17**, 17785-17789 (2015).

4 Artyushkova, K., Serov, A., Rojas-Carbonell, S. & Atanassov, P. Chemistry of Multitudinous Active Sites for Oxygen Reduction Reaction in Transition Metal–Nitrogen–Carbon Electrocatalysts. *The Journal of Physical Chemistry C* **119**, 25917-25928, doi:10.1021/acs.jpcc.5b07653 (2015).

5 Luo, F. *et al.* P-block single-metal-site tin/nitrogen-doped carbon fuel cell cathode catalyst for oxygen reduction reaction. *Nature Materials* **19**, 1215-1223, doi:10.1038/s41563-020-0717-5 (2020).
