# Supplementary material for: Electrochemical carbonyl reduction on single-site M–N–C catalysts
Source: Commun Chem. 2023 Sep 30;6:212. doi: 10.1038/s42004-023-01008-y (PMC10542751; doi:10.1038/s42004-023-01008-y)
Supplement: Supplementary file 2 — Description of Additional Supplementary Files [file 42004_2023_1008_MOESM2_ESM.pdf]

Description of Additional Supplementary Files

File name: Supplementary Data 1

Description: Data of electrochemical measurements
